# Social Media Membership, Browsing, and Profile Updating in a Representative U.S. Sample: Independent and Interdependent Effects of Big Five Traits and Aging and Social Factors

**DOI:** 10.3389/fpsyg.2017.01122

**Published:** 2017-06-30

**Authors:** Tim Bogg

**Affiliations:** Department of Psychology, Wayne State UniversityDetroit, MI, United States

**Keywords:** Big Five, social media, representative sample, aging, physical limitations

## Abstract

Guided by cybernetic perspectives on personality, the present work used a representative sample of U.S. adults (*N* = 992) to examine Big Five personality traits and social and aging factors as predictors of social media network membership and past-month browsing/searching and profile updating among members. The results showed adults who were less extraverted and less neurotic and who reported greater physical limitations were less likely to be members. Moreover, extraverted adults without partners were more likely to be members than introverted adults without partners. Among members, the results showed extraverted and emotionally stable younger and older adults reported a similar frequency of profile updating. In contrast, older adults with all other combinations of extraversion and neuroticism showed a reduced frequency of profile updating compared to younger adults. The findings are discussed in terms of social media involvement as a response of a self-regulatory system of personality adaptation.

## Introduction

According to data from the Pew Internet Survey Project, approximately 65% of U.S. adults aged 18 years and older use a social media networking site (Perrin, 2015). Facebook continues to be the most popular site, but others, including LinkedIn and Twitter, show continued growth in popularity (Duggan et al., 2015). Increases in social media site use have been especially pronounced among older adults (aged 65 years and older), with more than 50% of online older adults reporting use of a social media site. Moreover, more than half of online adults report using multiple social media sites.

While teen and young adult use of Facebook remains an informative target of inquiry for social media researchers (cf. Mark and Ganzach, 2014), the trends noted above highlight the need to examine broader (i.e., not limited to Facebook) social media participation and usage trends across the aging continuum. The goal of the present study was to use a self-regulatory personality framework to examine the interplay among dispositional drives and aging (i.e., age, self-rated health, health-related physical limitations) and social (i.e., relationship status) factors in the prediction of social media network membership and past-month network browsing/searching and profile updating in a representative sample of U.S. adults.

### Social media participation as a partial response of a system of personality adaptation

Models of interdependencies among major traits (i.e., the Big Five; Goldberg, 1993) have received increasing theoretical and empirical attention in recent years (Hirsh et al., 2009; Van Egeren, 2009; Bogg and Vo, 2014; DeYoung, 2015). These models are derived from cybernetic feedback control theory, which describes how machines respond to various informational inputs to meet regulatory goals (Weiner, 1948; e.g., cruise control modulating speed in response to varying grades of road steepness). For personality adaptation, such models posit independent and interdependent functions for traits that result in internal and/or external responses that aid in the potentiation of self-regulatory goals. Responses by the personality system—whether fostered through independent or interdependent trait drives—are produced to facilitate the attainment of valued goals that are informed by the relevant trait drive or drives. For example, a group norm for greater accessibility through social media could serve as an input to the personality system. Given this eliciting input, it would be expected that individual differences in extraversion (outgoing vs. inhibited) should be associated with varying internal responses (e.g., enthusiasm for more social contact vs. dismissal of the norm) and external responses (e.g., joining many social media sites vs. rejecting social media invitations).

The conceptualization of social media involvement as a partial function of a cybernetic system of personality adaptation provides for theoretically-driven expectations of the influences of traits and life context inputs. As described below, cybernetic perspectives can help clarify and constrain possible independent and interdependent effects of traits and life context factors in the prediction of social media involvement. The expected relations can be divided between independent (main) and interdependent (interactive) effects of traits on membership, browsing/searching, and profile updating—in combination with specific life context inputs.

#### Independent big five trait influences on social media site membership

The relation of a major trait domain to a personality system response is posited to be a function of the drive for that trait domain. That is, in the absence of a life context input, a personality system response is an attempt to satisfy endogenous levels of drive for a trait.

One of the primary drives posited for extraversion is the regulation of approach-related actions and behaviors (Van Egeren, 2009; DeYoung, 2015). With this drive in mind, social media site membership would serve as another means (or response) that would satisfy a greater level of the drive for approaching and engaging the social environment. The primary drive ascribed to openness is exploring abstract or sensory information and stimuli (Van Egeren, 2009; DeYoung et al., 2012; DeYoung, 2015). In this case, social media site membership would serve as another means (or response) that would satisfy a greater level of the drive to explore abstract and sensory information. The primary drive ascribed to agreeableness is cooperation (Van Egeren, 2009; DeYoung, 2015). In the absence of life context inputs that might cue affiliative tendencies, greater endogenous levels of the drive for supportiveness and commiseration alone are not likely to contribute to the response of joining a social media site. Similarly, without the presence of defined tasks, contending environmental demands, or other inputs that signal the need for temporal forbearance, it is not expected that conscientiousness alone would be associated with social media site membership. Finally, in the absence of normatively threatening or alarming inputs that might prompt the error information signaling and processing functions ascribed to neuroticism (Carver and Scheier, 1990; Lahey, 2009; Van Egeren, 2009), it is not expected that individual differences in emotional stability alone would be associated with social media site membership. To summarize, it is expected that greater levels of extraversion and openness should be positively associated with social media site membership (cf. Correa et al., 2010)—a socially communicative forum with available multi-sensory experiences that is consistent with the drives ascribed to these traits.

#### Interdependent big five trait and aging and social context influences on social media site membership

As a complement to models of independent influences, models of interdependent influences accommodate trait interactions in combination with life context inputs. The life context factors (i.e., inputs) examined herein were age, self-rated health, health-related physical limitations, and relationship status.

In the present study, guidance regarding interdependent effects among traits and life context factors came from the expected independent trait effects (i.e., extraversion, openness) and from cybernetic perspectives on the organization of Big Five traits as subcomponents of a self-regulatory system that responds to system inputs. Based on factor-analytic findings for the Big Five and Cybernetic Big Five Theory, two meta-traits—stability and plasticity—are thought to represent superordinate mechanisms that monitor, control, and adapt system functioning to enable the actuation of regulatory goals (Digman, 1997; Hirsh et al., 2009; DeYoung, 2015). Stability represents components of neuroticism, conscientiousness, and agreeableness, providing means by which error and goal control, monitoring, and detection related to system inputs (internal and external factors and circumstances) can be implemented. Plasticity represents components of extraversion and openness, providing means by which responses to system inputs can be implemented. In terms of interdependence, stability and plasticity and their respective traits are posited to interact in order to maintain the pursuit of valued goals and to enable adaptation in the context of life circumstances (DeYoung, 2015). By dividing the five traits in this complementary way, a self-regulatory cybernetic approach offers the additional benefit of constraining the number of possible interactive trait effects under consideration.

Given the error signaling function of neuroticism, it was expected that normatively threatening life circumstances (i.e., older age, poorer health, greater limitations, and being single) among neurotic and extraverted/open individuals would be associated with a greater probability of being a member of a social media site compared to stable and introverted/incurious individuals. In the predicted three-way interactions among neuroticism, extraversion/openness, and aging and social factors, adaptation for neurotic and extraverted/open younger or single individuals could be made manifest, in part, by responses that might increase social/stimuli exposure (i.e., by joining a social media site). By contrast, adaptation for emotionally stable and introverted/incurious individuals in poor health or with physical limitations could be made manifest by responses that conserve resources and eschew social/stimuli exposure, including social media sites.

#### Independent big five trait influences on social media site browsing/searching and profile updating among network members

Given the search functionality of social media sites, greater levels of the exploratory drive posited for openness should be associated with greater browsing/searching of social media sites among network members, even in the absence of system inputs (e.g., invitation to contact a network member). In contrast, the drives associated with extraversion (i.e., approach), neuroticism (i.e., error monitoring), conscientiousness (i.e., error/goal control), and agreeableness (i.e., cooperation) would seem insufficient to prompt browsing/searching in the absence of system inputs.

Compared to the exploratory behavior of browsing/searching, profile updating is a set of behaviors characterized by the production of self-disclosing communications and representations. As such, greater levels of approach drive associated with extraversion are expected to be associated with a greater frequency of profile updating among network members, which might be perceived as yet another means of projecting oneself into the social world. In contrast, the drives associated with neuroticism (i.e., error monitoring), conscientiousness (i.e., error/goal control), agreeableness (i.e., cooperation), and openness (i.e., exploratory) would seem insufficient to prompt profile updating in the absence of system inputs.

#### Interdependent big five trait influences on social media site browsing/searching and profile updating among network members

Given the prediction of an independent influence of greater levels of openness for browsing/searching, this trait becomes a primary candidate for inclusion in a larger interdependent model of personality adaptation in relation to browsing/searching of social media sites.

As was predicted for social media site membership, the experience of normatively threatening life circumstances (i.e., older age, poorer health, greater limitations, and being single) among neurotic and open individuals was expected to be associated with a greater frequency of browsing/searching a site compared to stable and incurious individuals. In the predicted three-way interactions for neuroticism, openness, and aging and social factors, adaptation for neurotic and open younger or single individuals could be made manifest, in part, by increased probing actions through various means (e.g., searching for other young singles via social media). In contrast, adaptation for stable and incurious individuals in poor health or with physical limitations (those predicted to be less likely to join networks in the first place) is less likely to be made manifest by such probing actions.

Similarly, it seems likely that the experience of normatively threatening life circumstances (i.e., older age, poorer health, greater limitations, and being single) among neurotic and extraverted individuals would be associated with a greater frequency of profile updating compared to stable and introverted individuals. In the predicted three-way interactions for neuroticism, extraversion, and aging and social factors, adaptation for neurotic and extraverted younger or single individuals could be made manifest, in part, by increased self-disclosing actions through various means (e.g., frequent profile updating), whereas personality system adaptation for stable and introverted individuals in poor health or with physical limitations (again, those predicted to be less likely to join networks in the first place) are less likely to be made manifest by self-disclosing actions.

## Materials and methods

### Participants

The data collection (commissioned by the author) was conducted using the Web-enabled KnowledgePanel®, a probability-based national panel designed to be representative of the U.S. adult population (Note: Complete data and documentation are expected to be publicly available at osf.io/a5ny2 by 12/2018). Initially, participants were chosen systematically by a random selection of telephone numbers and residential addresses. Persons in selected households were then invited by telephone or by mail to participate. For individuals who agreed to participate, but did not have Internet access, GfK provided a laptop and ISP connection at no cost. People who had computers and Internet service were allowed to complete the survey using their own equipment. Panelists received unique logins for completing the online survey (completion rate: 63.4%). For a more detailed explanation and justification for the representativeness of the KnowledgePanel® sample, readers can follow this link: http://www.knowledgenetworks.com/ganp/docs/KnowledgePanelR-Statistical-Methods-Note.pdf. The study was approved by Wayne State University's Institutional Review Board with exempt status. Participants were consented by GfK as members of the KnowledgePanel®. To encourage and incentivize participation and survey completions, GfK offers raffles and sweepstakes with cash rewards and prizes to be won. In the present study, participants (*N* = 992) ranged from 18 to 88 years of age, with a mean age of 49.57 years (*SD* = 17.13 years). The sample was sex-balanced (50.7% females) and the majority of the participants were White, Non-Hispanic (66.7%).

### Assessment materials

#### Relationship status, education, income, employment status, and household internet access

Relationship status was assessed with a single dichotomously scored item (no partner/with partner: 0 = single, widowed, divorced, separated, or never married; 1 = married or living with partner). Highest level of education attained was assessed using scores from one of 14 categories (1 = No formal education, 14 = Professional or Doctorate degree). Income was assessed using scores from one of 19 categories (1 = < $5,000; 19 = $175,000 or more). Employment status was assessed with a single item (1 = employed, 0 = not working due to temporary layoff, retirement, disability, other, or looking for work). Household Internet access was assessed using a dichotomous variable indicating the presence or absence of Internet access in participants' residences (1 = Yes, 0 = No).

#### Self-rated health

Self-rated health was assessed with a single item using a five-point scale (In general, would you say your health is? 1 = Poor, 5 = Excellent).

#### Health-related physical limitations

Physical limitations were assessed with 10 items from the SF-36 (Ware and Sherbourne, 1992) used to measure limitations in daily physical activities resulting from health problems (e.g., lifting or carrying groceries, climbing stairs, and bending, kneeling, or stooping; α = 0.95). Health-related physical limitations are commonly included in measures of health-related quality of life, a construct that has shown relations to subjective well-being and that is intended to capture a range of perceptions about functional status that go beyond self-rated health and/or objective/diagnostic indicators of morbidity (McHorney, 1999; Garrido et al., 2013). In this way, greater health-related physical limitations serve as an indicator of perceptions about lower functional well-being.

The limitations variable showed evidence of a skewed distribution. To address this, raw scores were Blom-transformed in the complete sample and separately for the subsample of social media site members. A Blom transformation rank orders raw scores (resolving tie scores by using the mean of the contested ranks) and transforms the ranks to *z* scores using the normal distribution. Research examining various types of transformation types showed a Blom transformation of symptom count data (i.e., skewed data similar to the limitations data) resulted in a more accurate selection of a true model from a set of alternative models (van den Oord et al., 2000). In the present work, the use of raw vs. Blom-transformed limitations scores did not produce a different pattern of effects in the models for social media site membership. However, the use of raw limitations scores in the browsing/searching and profile updating linear regression models resulted in non-significant independent and interdependent effects compared to models using the Blom-transformed limitations variable. This is likely due to the greater amount of skew and kurtosis for the limitations variable in the subsample of social media site members (compared to the complete sample), and the greater correction the transformation produced for the limitations variable in this subset of data.

#### Big five inventory (BFI)

The well-validated 44-item BFI was used to assess five broad domains of personality traits (John et al., 2008). All items were rated using a five-point scale (1 = Disagree Strongly, 5 = Agree Strongly). A nine-item scale was used to assess extraversion (e.g., “is outgoing, sociable”; α = 0.81). An eight-item scale was used to assess neuroticism (e.g., “gets nervous easily”; α = 0.85). A ten-item scale was used to assess openness (e.g., “is curious about many different things”; α = 0.80). A nine-item scale was used to assess conscientiousness (e.g., “does a thorough job”; α = 0.81). An eight-item scale was used to assess agreeableness (e.g., “is helpful and unselfish with others”; α = 0.81).

#### Social media network membership, network browsing/searching, and network profile updating

Social media network membership was assessed with a single item (“Do you belong to a social media network, such as Facebook, Twitter, LinkedIn, Google+, or MySpace?”; 0 = No, 1 = Yes). Additionally, network members were asked to rate how often during the past month they “browsed or searched one (or more) of the social media networks for which you are a member?” (0 = Never, 1 = Once in past month, 2 = Weekly, 3 = A few times per week, 4 = Daily, 5 = More than once per day). Network members were also asked to rate how often during the past month they “edited or created information for your personal page or feed (e.g., changed/updated profile or status information) on one (or more) of the social networks for which you are a member?” (0 = Never, 1 = Once in past month, 2 = Weekly, 3 = A few times per week, 4 = Daily, 5 = More than once per day).

### Analytic approach

Power analyses using G*Power 3.1.5 (Faul et al., 2009) showed that under the assumption of small main and interaction effects (f^2^ = 0.04), α = 0.05, 1-β = 0.85, and 19 predictors, a sample size of 585 participants would be required. The acquired social media site member subsample size of 630 suggests a relatively high degree of confidence in detecting small effects, while the total sample size (*N* = 992) suggests confidence in detecting even smaller effects for the analyses examining social media site membership.

Sampling weights were used in all analyses to modify the sample characteristics to be representative of the U.S. population. This weighting procedure adjusted for survey non-response, as well as non-coverage, under- or over-sampling, or participant demographic factors (sex, age, race/ethnicity, education, census region, household income, residence in a metropolitan area, and Internet access). Correlational analyses were used to examine the strength and direction of associations among the study variables.

**Figure 1:**
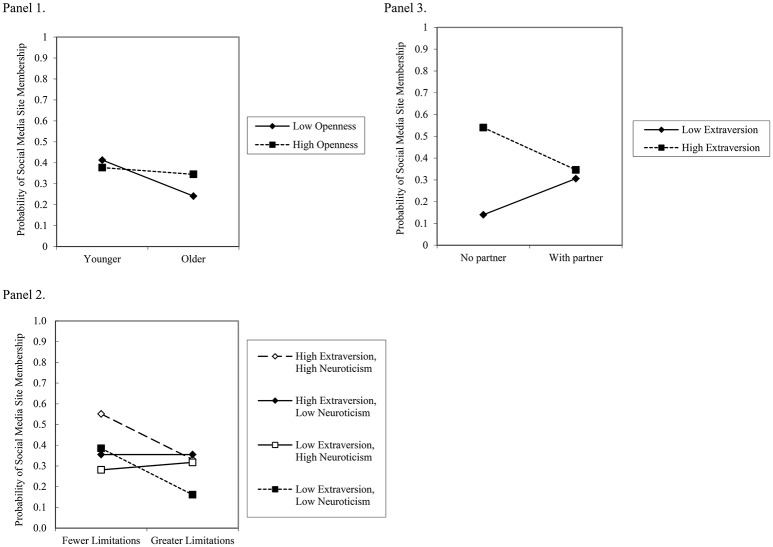
**Panel 1** depicts the two-way interaction between high and low (±1 standard deviation) age and openness in the prediction of social media site membership (simple slopes for low openness, *b* = −0.09, SE = 0.02, *p* < 0.01; high openness; *b* = −0.02, SE = 0.02, *ns*). **Panel 2** depicts the three-way interaction among high and low (±1 standard deviation) levels of neuroticism, extraversion, and health-related physical limitations in the prediction of social media site membership (simple slopes for high extraversion, high neuroticism, *b* = −0.11, SE = 0.04, *p* < 0.05; high extraversion, low neuroticism, *b* = 0, SE = 0.03, *ns*; low extraversion, high neuroticism, *b* = 0.02, SE = 0.03, *ns*; low extraversion, low neuroticism, *b* = −0.11, SE = 0.04, *p* < 0.05). **Panel 3** depicts the two-way interaction between high and low (±1 standard deviation) levels of extraversion and partnered status in the prediction of social media site membership (simple slopes for low extraversion, *b* = 0.08, SE = 0.05, *ns*; high extraversion; *b* = −0.10, SE = 0.05, *p* < 0.05).

Logistic regression analyses are typically recommended for dichotomous outcomes, such as social media site membership. However, the choice of logistic vs. linear regression can be rendered as a function of the features of the dataset, the specific variable in question, and the intent of the analyses. Using a variety of simulations, Hellevik (2009) identified circumstances under which the choice of logistic regression vs. linear regression can be equivocal, as well as circumstances under which linear regression would be preferred for dichotomous outcomes. For the present work, the use of a larger sample size, an event occurrence rate located away from the extremes, an interest in modeling causal (vs. predictive) effects, and, especially, the ease of interpretation suggest the appropriateness of a preference for using linear regression for examining social media site membership. Despite this preference, for sensitivity purposes, parallel logistic regression analyses were conducted for the social media site membership models depicted in **Table 3** and Figure 1. The results of these analyses—reported in Supplementary Table 1—showed the odds ratios matched the direction of effects and pattern of statistical significance of the linear regression effects of the more accessible probability weights reported herein. Consequently, two sets of linear regression models were used for the independent and interdependent effects of the Big Five traits and aging and social factors on social media site membership. The first set of models simultaneously examined the associations of (1) background (including sex, white vs. non-white, education, household income, and household Internet access), (2) aging and social factors (age, self-rated health, limitations, single vs. partnered), (3) Big Five personality traits, and, specific to the social media membership, (4) the interactions among neuroticism, extraversion, and aging and social factors on social media site membership. The second set of models included the first three components of the first set of models, but differed in its examination of the interactions among neuroticism, openness, and aging and social factors on social media site membership. Consistent with suggestions by Jaccard and Turrisi (2003), non-dichotomous variables were standardized (i.e., z-scored) to aid in comparing effects using differing response scales, separately for the complete sample and the subsample of social media site members. Simple slopes analyses were conducted using calculators provided by Preacher et al. (2006).

The browsing/searching and profile updating variables were assessed using ordinal (rather than continuous) scales. Nonetheless, linear regression analyses, rather than ordinal regression analyses, were used for modeling these variables. Simulation work using extremely non-normal data showed large-sample (*N* > 500) linear regression approaches were robust, even to substantial deviations from normality (Lumley et al., 2002). In the present study, skewness and kurtosis were not substantial for the browsing/searching and profile updating variables. Consequently, one set of linear regression models was used to examine the independent and interdependent effects of the Big Five traits and aging and social factors on browsing/searching among social media site members. The models examined the associations of (1) background, (2) aging and social factors, (3) Big Five personality traits, and, specific to browsing/searching, (4) the interactions among neuroticism, openness, and aging and social factors on social media site membership. Finally, one set of linear regression models was used to examine the independent and interdependent effects of the Big Five traits and aging and social factors on profile updating among social media site members. The models simultaneously examined the associations of (1) background, (2) aging and social factors, (3) Big Five personality traits, and, specific to profile updating, (4) the interactions among neuroticism, extraversion, and aging and social factors on social media site membership.

## Results

### Descriptive statistics and correlational analyses

Table 1 displays the descriptive statistics for the study variables. Approximately 64% of the sample reported being a member of one or more social media network sites, which is consistent with a recent estimate of 65% by the Pew Research Center (Perrin, 2015). Among members, the average reported monthly frequency of browsing/searching was more than a few times per week, but less than daily. Among members, the average reported monthly frequency of profile updating was more than once per month, but less than weekly.

**Table 1:** Descriptive statistics for study variables (*N* = 992).

**Study variable**	**Mean (SD) or %**
Sex (% female; 0 = Male, 1 = Female)	50.7%
Age (years)	49.57 (17.13)
Ethnicity (% white)	66.7%
Education (1 = No formal education, 14 = Professional/Doctorate degree)	10.16 (1.97)
Household income (1 = <$5, 000, 19 = $175, 000 or greater)	11.63 (4.58)
Working (yes/no)	54.2%
Household Internet access (yes/no)	75%
Married/Living with a partner (yes/no)	62.9%
Health status (1 = poor, 5 = excellent)	3.33 (0.91)
Health-related physical limitations (0–20)	3.74 (5.28)
Extraversion (1–5)	3.10 (0.75)
Neuroticism (1–5)	2.75 (0.79)
Conscientiousness (1–5)	3.84 (0.64)
Agreeableness (1–5)	3.83 (0.68)
Openness (1–5)	3.37 (0.61)
Social media network member (yes/no)	63.5%
Among social media members, site browsing frequency (0 = never, 5 = more than once per day)	3.21 (1.53) Never (0) = 7.3% Once per month (1) = 9.1% Weekly (2) = 14.7% A few times per week (3) = 19% Daily (4) = 25.8% More than once per day (5) = 24%
Among social media members, profile updating frequency (0 = never, 5 = more than once per day)	1.41 (1.37) Never (0) = 33.6% Once per month (1) = 26.9% Weekly (2) = 14.4% A few times per week (3) = 16.1% Daily (4) = 6.7% More than once per day (5) = 2.4%

Above the dashed line of Table 2 are the correlations among the study variables for the complete sample (excluding the browsing/searching and profile updating variables). Below the dashed line are correlations among the study variables for the subsample that reported being a member of a social media network site, including the browsing/searching and profile updating variables.

**Table 2:** Correlations among study variables.

	**1**	**2**	**3**	**4**	**5**	**6**	**7**	**8**	**9**	**10**	**11**	**12**	**13**	**14**	**15**	**16**	**17**
1.Sex	–																
2. Age	0.04	–															
3. Ethnicity (White)	−0.01	0.15^*^	–														
4. Education	0.03	−0.02	0.14^*^	–													
5. Household income	−0.06	−0.04	0.24^*^	0.41^*^	–												
6. Working	−0.12^*^	−0.29^*^	0.12^*^	0.19^*^	0.30^*^	–											
7. Household Internet access	0.16^*^	−0.14^*^	0.08^*^	0.25^*^	0.45^*^	0.15^*^	–										
8. Married/Living with partner	0.01	0.13^*^	0.08^*^	0.17^*^	0.29^*^	0.10^*^	0.15^*^	–									
9. Health status	−0.08^*^	−0.12^*^	0.02	0.24^*^	0.23^*^	0.12^*^	0.11^*^	0.03	–								
10. Health-related limitations	0.12	0.21^*^	−0.06^†^	−0.22^*^	−0.28^*^	−0.32^*^	−0.20^*^	−0.09^*^	−0.43^*^	–							
11. Extraversion	−0.02	−0.07^†^	−0.07^†^	−0.02	0.10^*^	0.10^*^	0.03	0.07^†^	0.23^*^	−0.08^*^	–						
12. Neuroticism	0.16^*^	−0.13^*^	0.09^*^	−0.08	−0.10^*^	−0.05	−0.04	−0.11^*^	−0.31^*^	0.12^*^	−0.26^*^	–					
13. Conscientiousness	0.06	0.20^*^	0.02	0.12^*^	0.12^*^	0.07^†^	0.05	0.12^*^	0.19^*^	−0.15^*^	0.21^*^	−0.38^*^	–				
14. Agreeableness	0.06	0.18^*^	0.04	0.10^*^	0.06^*^	−0.03	0.04	0.04	0.15^*^	−0.15^*^	0.17^*^	−0.41^*^	0.48^*^	–			
15. Openness	−0.08^*^	0.00	−0.05	0.12^*^	−0.02	−0.04	0.08^*^	−0.09^*^	0.22^*^	−0.13^*^	0.30^*^	−0.14^*^	0.24^*^	0.15^*^	–		
16. Social media site member	0.12^*^	−0.18^*^	0.06	0.19^*^	0.15^*^	0.19^*^	0.25^*^	0.02	0.04	−0.14^*^	0.10^*^	0.07^†^	−0.01	0.01	0.07^†^	–	
17. Members: Browsing/searching	0.05	−0.16^*^	−0.01	−0.03	0.00	−0.01	0.07	0.05	−0.04	−0.04	0.04	0.02	−0.06	−0.06	−0.02	–	–
18. Members: Profile updating	0.00	−0.28^*^	−0.06	−0.05	−0.03	0.10^†^	0.07	−0.02	−0.07	0.01	0.13^*^	0.00	−0.12^*^	−11^*^	0.05	–	0.52^*^

*Correlations above the dashed line were computed based on data from the complete sample, excluding browsing/searching and profile updating variables (N = 992). Correlations below the dashed line were computed based on data from the subsample of participants that reported being members of social media sites for the browsing/searching and profile updating variables (N = 630). For sex, male = 0, female = 1*.

* *p < 0.01*,

† *p < 0.05*.

The sample-wide correlational results showed women, younger adults, adults with greater education and income, employed adults, adults with household Internet access, adults with fewer health-related physical limitations, more extraverted adults, more neurotic adults, and more open adults were more likely to be members of social media sites. Among network members, younger adults, adults with household Internet access, and adults who reported greater past-month profile updating were more likely to report more frequent searching/browsing of networks during the past month. Also among network members, younger adults, employed adults, more extraverted adults, less conscientiousness adults, less agreeable adults, and those adults who reported greater past-month searching/browsing were more likely to report greater past-month profile updating.

### Regression models for social media site membership

Consistent with expectations based on the proposed self-regulatory approach drive of extraversion, and controlling for background factors, higher scores on extraversion were associated with a greater probability of being a member of a social media site (see Table 3). Although greater openness and neuroticism showed positive associations with membership in the correlational analyses, when controlling for background factors, neither trait was associated with social media site membership in the regression analyses. Among the aging and social factors, only younger age was consistently associated with a greater probability of membership when controlling for other effects.

Table 3Additive and interactive effects of extraversion, neuroticism, openness, age, health-related physical limitations, and relationship status on social media site membership (*N* = 992).

**Table:** 

	**Weights of probability (out of 1) of social media site membership**		
	**Model 1 (*R* = 0.402) β | B (95% CI)**	**Model 2 (*R* = 0.406) β | B (95% CI)**	**Model 3 (*R* = 0.406) β | B (95% CI)**
1.Sex	0.14^*^ | 0.13 (0.07, 0.19)	0.14^*^ | 0.14 (0.08, 0.20)	0.14^*^ | 0.14 (0.07, 0.19)
2.Age	−0.11^*^ | −0.05 (−0.08, −0.02)	−0.11^*^ | −0.05 (−0.08, −0.02)	−0.11^*^ | −0.05 (−0.09, −0.02)
3.Ethnicity	0.03 | 0.03 (−0.03, 0.09)	.03 | 0.03 (−0.03, 0.10)	0.04 | 0.04 (−0.02, 0.10)
4.Education	0.12^*^ | 0.03 (0.01, 0.05)	0.13^*^ | 0.07 (0.03, 0.10)	0.15^*^ | 0.08 (0.04, 0.11)
5.Household income	−0.03 | −0.00 (−0.01, 0.00)	−0.04 | −0.02 (−0.05, 0.02)	−0.05 | −0.02 (−0.06, 0.01)
6.Working	0.11^*^ | 0.10 (0.04, 0.17)	0.10^*^ | 0.09 (0.03, 0.16)	0.10^*^ | 0.10 (0.03, 0.16)
7.Household Internet access	0.20^*^ | 0.22 (0.14, 0.29)	0.20^*^ | 0.22 (0.15, 0.29)	0.20^*^ | 0.22 (0.15, 0.30)
8.Married/Living with a partner	−0.02 | −0.02 (−0.08, 0.05)	−0.02 | −0.02 (−0.07, 0.05)	−0.01 | −0.01 (−0.07, 0.06)
9.Health status	−0.06 | −0.03 (−0.07, 0.01)	−0.06 | −0.03 (−0.06, 0.01)	−0.07 | −0.03 (−0.07, 0.00)
10.Health-related physical limitations	−0.07 | −0.04 (−0.08, 0 00)	−0.09^†^ | −05 (−0.09, −0.01)	−0.07 | −0.04 (−0.08, 0.00)
11.Extraversion	0.11^*^ | 0.07 (0.03, 0.11)	0.12^*^ | 0.06 (0.02, 0.09)	0.23^*^ | 0.11 (0.06, 0.16)
12.Neuroticism	0.07 | 0.04 (−0.00, 0.09)	0.06 | 0.03 (−0.00, 0.07)	0.09 | 0.04 (−0.01, 0.09)
13.Conscientiousness	−0.05 | −0.02 (−0.09, 0.02)	−0.04 | −0.02 (−0.06, 0.01)	−0.04 | −0.02 (−0.05, 0.02)
14.Agreeableness	0.04 | 0.02 (−0.02, 0.08)	0.04 | 0.02 (−0.02, 0.05)	0.03 | 0.01 (−0.02, 0.05)
15.Openness	0.04 | 0.02 (−0.02, 0.08)	0.04 | 0.02 (−0.02, 0.05)	0.04 | 0.02 (−0.01, 0.05)

**Table:** 

**Model 1**	**β | B (95% CI)**	**Model 2**	**β | B (95% CI)**	**Model 3**	**β | B (95% CI)**
N × O	0.04 | 0.02 (−0.01, 0.05)	N × E	0.03 | 0.02 (−0.01, 0.04)	N × E	−0.03 | −0.01 (−0.05, 0.03)
N × Age	−0.04 | −0.02 (−0.05, 0.01)	N × Limitations	0.01 | 0.01 (−0.02, 0.04)	N × With partner	−0.02 | −0.01 (−0.07, 0.05)
O × Age	0.07^†^ | 0.04 (0.01, 0.07)	E × Limitations	−0.01 | −0.01 (−0.04, 0.03)	E × With partner	−0.14^*^ | −0.09 (−0.15, −0.03)
N × O × Age	0.02 | 0.01 (−0.02, 0.04)	N × E × Limitations	−0.11^*^ | −0.06 (−0.10, −0.03)	N × E × With partner	0.07 | 0.04 (−0.01, 0.10)

* p < 0.01,

† *p < 0.05 Regression models were constructed using simultaneous entry of all terms. Bs are interpretable in outcome units (probability (out of 1) of social media site membership). E, Extraversion; N, Neuroticism; O, Openness. For sex, male = 0, female = 1*.

It was expected that normatively threatening circumstances (i.e., older age, poorer health, greater limitations, and being single) among neurotic and extraverted/open individuals would be associated with a greater probability of being a social media site member compared to stable and introverted/incurious individuals.

#### Age and openness

A two-way interaction was observed between age and openness (see Figure 1, Panel 1). The form of the interaction shows the combination of low levels of openness, in combination with older age, was associated with the lowest probability of membership. By comparison, curious *or* incurious younger individuals showed no differences in the probability of membership.

#### Health-related physical limitations, neuroticism, and extraversion

A three-way interaction was observed among physical limitations, neuroticism, and extraversion (see Figure 1, Panel 2). The form of the interaction shows the combination of high levels of neuroticism and extraversion, in combination with fewer limitations, was associated with a greater probability of membership. By comparison, emotionally stable and introverted adults who reported greater limitations showed the lowest probability of membership. For adults with either the high-low or low-high combinations of neuroticism and extraversion, respectively, the probabilities of membership did not differ as a function of limitations.

#### Partner status and extraversion

A two-way interaction was observed between partner status and extraversion (see Figure 1, Panel 3). The form of the interaction shows extraverted single adults had a greater probability of membership than introverted single adults. By comparison, extraverted *or* introverted adults with partners showed no differences in the probability of membership.

### Regression models for past-month social media site browsing/searching by members

It was expected that greater levels of the exploratory drive posited for openness should be associated with greater browsing/searching of social media sites among network members. Contrary to expectations, openness was not associated with social media site browsing/searching by members (see Table 4). Among the aging and social factors, only younger age was consistently associated with more frequent past-month site browsing/searching by members.

Table 4Additive and interactive effects of neuroticism, openness, age, and health-related physical limitations on social media site browsing/searching frequency by network members (*N* = 630).

**Table:** 

	**Past-month frequency of social media site browsing/searching**	
	**Model 1 (*R* = 0.236) β | B (95% CI)**	**Model 2 (*R* = 0.247) β | B (95% CI)**
1.Sex	0.05 | 0.15 (−0.11, 0.41)	0.05 | 0.15 (−0.11, 0.41)
2.Age	−0.18^*^ | −0.28 (−0.41, −0.14)	−0.19^*^ | −0.29 (−0.43, −0.15)
3.Ethnicity	0.02 | 0.06 (−0.10, 0.16)	0.02 | 0.05 (−0.23, 0.33)
4.Education	−0.02 | −0.03 (−0.17, 0.11)	−0.02 | −0.02 (−0.16, 0.12)
5.Household income	−0.02 | −0.03 (−0.19, 0.13)	−0.02 | −0.02 (−0.18, 0.13)
6.Working	−0.06 | −0.18 (−0.46, 0.09)	−0.06 | −0.19 (−0.09, 0.45)
7.Household Internet access	0.09 | 0.35 (−0.01, 0.71)	0.09 | 0.36 (0.00, 0.72)
8.Married/Living with a partner	0.07 | 0.22 (−0.05, 0.48)	0.06 | 0.18 (−0.09, 0.45)
9.Health status	−0.07 | −0.11 (−0.25, 0.04)	−0.07 | −0.10 (−0.25, 0.04)
10.Health−related physical limitations	−0.02 | −0.03 (−0.21, 0.14)	0.01 | 0.01 (−0.16, 0.19)
11.Extraversion	0.05 | 0.08 (−0.06, 0.22)	0.05 | 0.07 (−0.07, 0.21)
12.Neuroticism	−0.05 | −0.07 (−0.22, 0.07)	−0.04 | −0.07 (−0.21, 0.08)
13.Conscientiousness	0.02 | 0.03 (−0.11, 0.17)	0.01 | 0.02 (−0.12, 0.16)
14.Agreeableness	−0.06 | −0.09 (−0.23, 0.06)	−0.06 | −0.09 (−0.24, 0.05)
15.Openness	0.01 | 0.01 (−0.13, 0.15)	0.00 | 0.00 (−0.13, 0.14)

**Table:** 

**Model 1**	**β | B (95% CI)**	**Model 2**	**β | B (95% CI)**
N × O	−0.02 | −0.02 (−0.14, 0.10)	N × O	−0.02 | −0.02 (−0.14, 0.09)
N × Age	−0.09^†^ | −0.14 (−0.26, −0.01)	N × Limitations	−0.09^†^ | −0.16^†^ (−0.32, −0.01)
O × Age	0.01 | 0.02 (−0.11, 0.15)	O × Limitations	0.06 | 0.11 (−0.04, 0.25)
N × O × Age	0.07 | 0.09 (−0.03, 0.21)	N × O × Limitations	0.09^†^ | 0.14 (0.01, 0.28)

* p < 0.01,

† *p < 0.05 Regression models were constructed using simultaneous entry of all terms. Bs are interpretable in outcome units (past-month browsing/searching frequency). N, Neuroticism; O, Openness. For sex, male = 0, female = 1*.

As was predicted for social media site membership, it was expected that the experience of normatively threatening life circumstances (i.e., older age, poorer health, greater limitations, and being single) among neurotic and open individuals would be associated with a greater frequency of browsing/searching of a site compared to stable and incurious individuals.

#### Age and neuroticism

**Figure 2:**
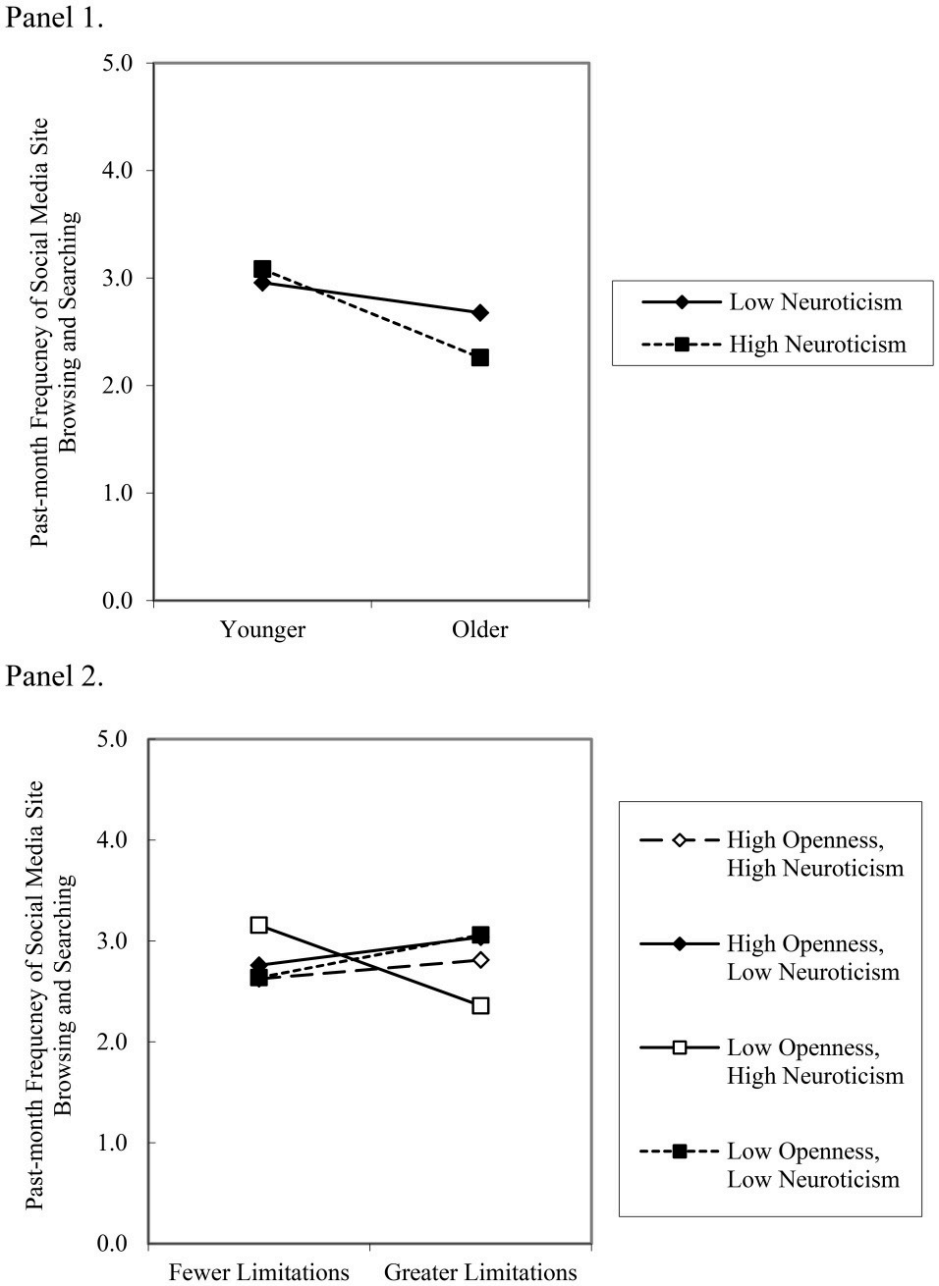
**Panel 1** depicts the two-way interaction between high and low (±1 standard deviation) age and neuroticism in the prediction of past-month social media site browsing/searching by network members (simple slopes for low neuroticism, *b* = −0.14, SE = 0.09, *ns*; high neuroticism; *b* = −0.41, SE = 0.09, *p* < 0.01). **Panel 2** depicts the three-way interaction between high and low (±1 standard deviation) levels of health-related physical limitations, neuroticism, and openness in the prediction of past-month social media site browsing/searching by network members (simple slopes for high openness, high neuroticism, *b* = 0.09, SE = 0.16, *ns*; high openness, low neuroticism, *b* = 0.21, SE = 0.17, *ns*; low openness, high neuroticism, *b* = 0.14, SE = 0.14, *ns*; low openness, low neuroticism, *b* = −0.40, SE = 0.16, *p* < 0.05).

A two-way interaction was observed between age and neuroticism (see Figure 2, Panel 1). The form of the interaction shows the combination of high levels of neuroticism, in combination with older age, was associated with the least frequent browsing/searching among social media site members. By comparison, emotionally stable *or* anxious younger adults showed no differences in browsing/searching frequency.

#### Health-related limitations, neuroticism, and openness

A three-way interaction was observed among health-related limitations, neuroticism, and openness (see Figure 1, Panel 2). The form of the interaction shows neurotic and incurious adults with greater limitations reported less frequent browsing/searching than neurotic and incurious adults with fewer limitations. By comparison, emotionally stable and curious *or* neurotic and curious *or* emotionally stable and incurious adults showed no differences in browsing/searching frequency as a function of health-related limitations.

### Regression models for past-month social media site profile updating by members

Greater levels of the approach drive associated with extraversion were expected to be associated with a greater frequency of profile updating among network members. Consistent with the expectation for an independent effect, higher extraversion scores were associated with more frequent social media site profile updating by members (see Table 5). Among the aging and social factors, younger age and greater health-related physical limitations were consistently associated with greater profile updating by network members.

Table 5Additive and interactive effects of neuroticism, extraversion, age, and health-related physical limitations on social media site profile updating frequency by network members (*N* = 630).

**Table:** 

	**Past-month frequency of social media site profile updating**	
	**Model 1 (*R* = 0.419) β | B (95% CI)**	**Model 2 (*R* = 0.415) β | B (95% CI)**
1.Sex	0.02 | 0.04 (−0.18, 0.26)	0.02 | 0.06 (−0.16, 0.28)
2.Age	−0.34^*^ | −0.47 (−0.59, −0.35)	−32^*^ | −0.44 (−0.55, −0.32)
3.Ethnicity	−0.03 | −0.09 (−0.32, 0.15)	−0.03 | −0.10 (−0.33, 0.11)
4.Education	0.00 | 0.00 (−0.12, 0.12)	−0.01 | −0.01 (−0.13, 0.11)
5.Household income	−0.08 | −0.11 (−0.24, 0.03)	−0.07 | −0.09 (−0.23, 0.04)
6.Working	0.06 | 0.18 (−0.06, 0.41)	0.06 | 0.17 (−0.06, 0.40)
7.Household Internet access	0.11^†^ | 0.40 (0.10, 0.70)	0.12^*^ | 0.44 (0.13, 0.74)
8.Married/Living with a partner	0.03 | 0.09 (−0.13, 0.32)	0.02 | 0.05 (−0.17, 0.28)
9.Health status	−0.07 | −0.10 (−0.22, 0.03)	−0.08 | −0.10 (−0.23, 0.02)
10.Health-related physical limitations	0.11^†^ | 0.18 (0.03, 0.33)	0.11^†^ | 0.18 (0.03, 0.33)
11.Extraversion	0.11^†^ | 0.15 (0.04, 0.27)	0.12^*^ | 0.17 (0.05, 0.29)
12.Neuroticism	−0.09 | −0.12 (−0.25, 0.00)	−0.09^†^ | −0.13 (−0.25, −0.00)
13.Conscientiousness	−0.07 | −0.10 (−0.22, 0.02)	−0.07 | −0.09 (−0.21, 0.03)
14.Agreeableness	−0.06 | −0.09 (−0.21, 0.03)	0.07 | −0.10 (−0.22, 0.02)
15.Openness	0.06 | 0.08 (−0.04, 0.19)	0.07 | 0.10 (−0.01, 0.22)

**Table:** 

**Model 1**	**β | B (95% CI)**	**Model 2**	**β | B (95% CI)**
N × E	0.10^†^ | 0.13 (0.03, 0.24)	N × E	0.07 | 0.09 (−0.01, 0.20)
N × Age	−0.05 | −0.07 (−0.17, 0.04)	N × Limitations	−0.11^*^ | −0.18 (−0.31, −0.05)
E × Age	0.06 | 0.08 (−0.03, 0.19)	E × Limitations	0.05 | 0.09 (−0.05, 0.22)
N × E × Age	−0.12^*^ | −0.14 (−0.24, −0.05)	N × E × Limitations	−0.05 | −0.05 (−0.17, 0.08)

* p < 0.01,

† *p < 0.05 Regression models were constructed using simultaneous entry of all terms. Bs are interpretable in outcome units (past-month profile updating frequency). N, Neuroticism; E, Extraversion. For sex, male = 0, female = 1*.

It was expected that the experience of normatively threatening life circumstances (i.e., older age, poorer health, greater limitations, and being single) among neurotic and extraverted individuals would be associated with a greater frequency of profile updating (self-disclosure) compared to stable and introverted individuals.

#### Age, neuroticism, and extraversion

**Figure 3:**
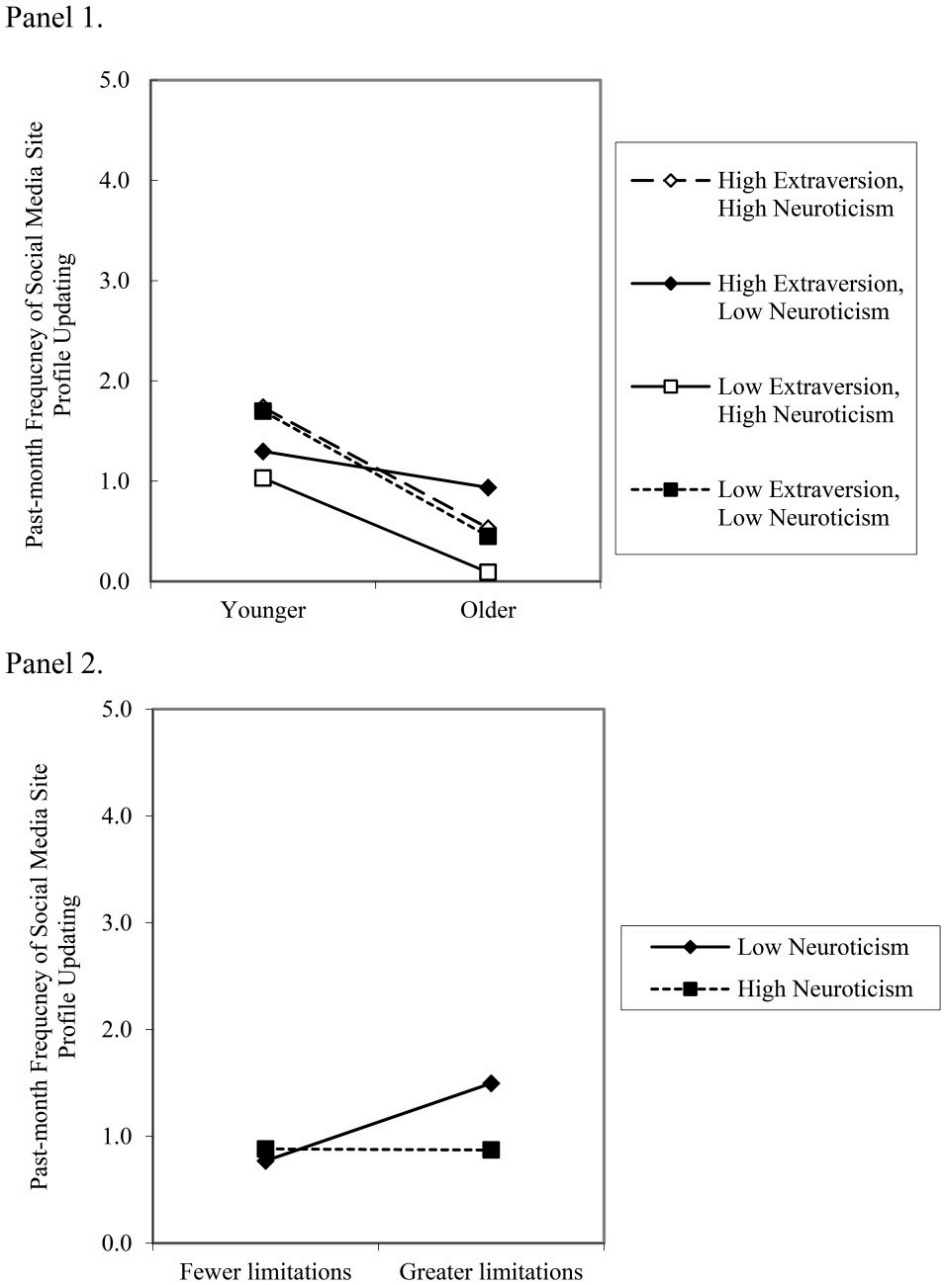
**Panel 1** depicts the three-way interaction between high and low (±1 standard deviation) age, neuroticism, and extraversion in the prediction of past-month social media site profile updating by network members (simple slopes for high extraversion, high neuroticism, *b* = −0.60, SE = 0.12, *p* < 0.01; high extraversion, low neuroticism, *b* = −0.18, SE = 0 09, *ns*; low extraversion, high neuroticism, *b* = −0.47, SE = 0.11, *p* < 0.01; low extraversion, low neuroticism, *b* = −0.62, SE = 0.12, *p* < 0.01). **Panel 2** depicts the two-way interaction between high and low (±1 standard deviation) levels of health-related physical limitations and neuroticism in the prediction of past-month social media site profile updating by network members (simple slopes for low neuroticism, *b* = 0.36, SE = 0.11, *p* < 0.01; high neuroticism; *b* = −0.01, SE = 0.10, *ns*).

A three-way interaction was observed between age, neuroticism, and extraversion (see Figure 3, Panel 1). The form of the interaction shows the combination of high levels of neuroticism and low levels of extraversion, in the context of older age, was associated with the lowest frequency of profile updating. Extraverted and anxious younger adults *or* introverted and emotionally stable younger adults showed the most frequent profile updating. By comparison, younger *or* older emotionally stable and extraverted adults showed no differences in profile updating frequency.

#### Health-related physical limitations and neuroticism

A two-way interaction was observed between health-related physical limitations and neuroticism (see Figure 3, Panel 2). The form of the interaction shows emotionally stable adults with fewer limitations reported less frequent profile updating compared to emotionally stable adults with greater limitations. By comparison, emotionally stable *or* neurotic adults with fewer limitations showed no differences in profile updating frequency.

## Discussion

Using data from a representative sample of U.S. adults, the goal of the present study was to investigate an independent and interdependent system of personality adaptation for social media site membership, browsing/searching, and profile updating by incorporating aging and social factors to discern life context influences on social media participation and use. Consistent with recent national survey data, socioeconomic resource factors, including education level, employment status, and household Internet access, were associated with a greater probability of being a member of a social media site (Duggan et al., 2015). Among the personality traits investigated, greater levels of extraversion were associated with a greater probability of being a member of a social media site, as well as more frequent profile updating by members. Moreover, several interactions among neuroticism, extraversion, openness, age, health-related physical limitations, and partner status provided evidence for interdependent effects of dispositional drives and aging and social factors on perceived adaptive responses of social media involvement in a system of personality adaptation. The implications of these results, as well as the strengths and limitations of the present study are discussed below.

### Approach and engagement drives predict social media site membership

The independent effect for extraversion speaks to the relatively robust relation of this trait domain to social media site membership—an effect that was similar in magnitude to those of age and education level. The effect for extraversion demonstrates the direct roles of drives for approaching and engaging with the social environment, even when that environment is not physical, but is mediated by means of digital communication and presentation. This result is similar to findings from a non-representative Australian sample of younger Internet users that focused on Facebook membership and only controlled for effects of other personality traits, as well as a non-representative U.S. sample that did not include effects of agreeableness, conscientiousness, employment status, household Internet access, health status, and physical limitations (Correa et al., 2010; Ryan and Xenos, 2011). In contrast to this prior research, the present work did not (expect nor) show conscientiousness to be a significant predictor of social media site membership. Moreover, although predicted, the present work did not show openness—a sensory and exploratory drive—to be an independent predictor of social media site membership.

In combination with the significant independent finding for extraversion, these results suggest—in the absence of system other inputs—the role of the plasticity mechanism of an interdependent model of personality adaptation for social media site membership is primarily informed by extraversion, not openness.

### Approach instability characterizes interdependent trait and aging and social effects on social media site membership

Consistent with neuroticism's regulatory role of signaling potential system instability, the results showed adults high in neuroticism and extraversion—high approach instability—with fewer physical limitations were the most likely to be members of social media sites, as compared to adults with greater physical limitations who were low on neuroticism and extraversion—low approach instability. It is notable that adults with greater physical limitations who also were high on approach instability were as likely to be members of social media sites as adults with moderate levels of approach instability who had fewer physical limitations. With regard to social media site membership, high approach instability would seem to serve a compensatory role in the context of greater physical limitations, offsetting possible barriers to participation through a stronger sensitivity for and putatively adaptive response to a potential opportunity for engagement in the social world. In a similar vein, greater levels of exploratory drive seem to compensate for effects of older age, where probabilities of social media site membership across age were quite similar for adults with greater levels of openness, but lower for incurious older adults.

Although not involving the internal error alarm of greater neuroticism, the system input of being without a partner seems to serve an analogous role for participation in social media when considered in combination with a greater approach drive. Compared to an adult without a partner who had a greater dispositional drive for approach and engagement, an introverted adult without a partner had a lower probability of being a member of a social media site. Without an external cue signaling the absence of access to a form of engagement with the social world, adults with partners, whether high or low on drives for approach and engagement, showed no differences in the probability of social media site membership.

### Alarm sensitivity characterizes interdependent trait and aging effects on browsing/searching among social media site members

Although an independent effect on browsing/searching was predicted for the exploratory drive of openness, the results showed no independent effect for this trait or any of the other Big Five traits. The null finding for openness is inconsistent with effects reported by Ryan and Xenos (2011), which showed greater levels of openness were associated with news and information feature usage in a younger sample of Australian Facebook members. The null finding also is somewhat at odds with prior work showing openness to be associated with a greater frequency of health-related Internet searches (Bogg and Vo, 2014). In the present work, the lack of a predictive effect for openness may be attributable to the combined assessment of browsing and searching into a single response, not limiting social media to Facebook, and/or the use of a representative sample of adults.

In contrast to the dearth of independent trait effects, two interdependent effects for neuroticism and aging factors on browsing/search by social media site members were found in the present study. The pattern of these interdependent effects can be characterized as alarm sensitivity, where a strong error alerting drive of neuroticism and a weak exploratory drive of openness in the contexts of older age or greater physical limitations were associated with an attenuation of browsing/searching compared to neurotic, emotionally stable, or curious adults who were younger or experiencing few, if any, physical limitations. Although speculative, the less frequent browsing/searching associated with alarm sensitivity may be a function of a reduced perception of the value of browsing/searching as a response for the personality system. Regulatory adaptation in the context of aging or lower functional status among anxious (and incurious) adults may not be served by more frequent perusal of social media content compared to emotionally stable and/or open adults with lower functional status, for whom more frequent perusal of social media content would seem to have greater perceived adaptive value.

### Greater approach and engagement predict profile updating among social media site members

As was the case with social media site membership, the independent effect for extraversion on profile updating demonstrates the roles of drives for approaching and engaging the social world, even in a forum for sociality that is electronically-mediated.

#### Approach instability characterizes interdependent trait and aging effects on profile updating among social media site members

Consistent with neuroticism's regulatory role of signaling potential system instability, the results showed older and younger adults low in neuroticism and high in extraversion—high approach, low instability—showed no differences in the frequency of profile updating, whereas all other approach instability combinations showed a reduced frequency of profile updating for older adults. These findings suggest the high-approach-low-instability configuration might serve a compensatory role where more frequent profile updating might be perceived as an adaptive response by older extraverted and stable adults who may not recognize or feel threatened by ageist expectations regarding their aging status.

Profile updating frequency was equivalent for individuals with fewer limitations who were either low in error alerting drive (i.e., emotionally stable) *or* high in error alerting drive (i.e., neurotic). In contrast, a strong error alerting drive was associated with more frequent profile updating among individuals with greater limitations compared to individuals with fewer limitations and strong *or* weak error alerting drives. It may be the case that emotionally stable adults with lower functional status may experience less self-inhibition or self-consciousness, for which more frequent profile updating would be an additional (and perhaps more convenient) forum for self-disclosure and self-expression.

### Limitations, implications, and conclusions

The use of a representative U.S. sample—assessed on a set of background variables, aging and social factors, and personality traits—is a substantive advance in characterizing social media site participation and use. In addition, the framing of social media site membership, browsing/searching, and profile updating as part of an interdependent system of personality adaptation represents a novel application of a personological perspective to these recently invented social contexts and behaviors. Despite these strengths, the present study is not without limitations.

Aside from only using self-reports in the assessments, the primary point of concern of the present work is the broad assessment of social network membership and use, rather than examining specific networks and their (possibly differential) use. However, as was noted above, more than 50% of U.S. adults reported being members of multiple networks (Duggan et al., 2015), highlighting the need to examine predictors of overall membership and usage patterns across network boundaries. Moreover, browsing/searching and updating were measured using single items, whose precision and reliability cannot be assessed. Future work should attempt to delineate and aggregate more specific forms of browsing/searching and updating actions.

Most of the observed effects were small in size, but were robust to many other variables of interest. This pattern of small effects highlights the overdetermined nature of social media involvement. In a related vein, the unknown replicability of the interaction effects is a limitation, especially given that non-experimental three-way interaction effects can be spurious. Consequently, the three-way interaction patterns depicted in Figures 1–3 should be regarded as provisional and requiring replication using very similar sampling and measurement approaches. This cautionary note is especially relevant for the browsing/searching and updating interaction effects that involved the limitations variable. As noted above, the Blom-transformed limitations scores, but not the raw limitations scores, produced the significant effects depicted in Tables 4, 5 and Panels 2 of Figures 2, 3. Despite this discrepancy for browsing/searching and profile updating, the more robust limitations-related results for social media site membership suggest health-related quality of life warrants additional attention in the study of social media involvement.

Although the Big Five traits were assessed using a valid instrument, the scales are intended to provide broad representation of each trait. Narrower facets of extraversion (e.g., sociability, Goldberg, 1999; gregariousness, Costa and McCrae, 1992), neuroticism (e.g., stability Goldberg, 1999; anxiety, Costa and McCrae, 1992), and openness (e.g., imagination, Goldberg, 1999; actions; Costa and McCrae, 1992) might provide improved predictive utility. Especially as it relates to browsing and searching of social media sites by members—for which openness showed no independent effect—it may be the case that a narrower facet of openness, such as ingenuity or quickness (Goldberg, 1999), might be more relevant to such behaviors and produce larger effects.

Although the aging and social factors assessed in the present work pertained to functional capacity (age, physical limitations), well-being (self-rated health), and social connectedness (with/without a partner), they were not exhaustive. Moreover, an assessment of motives for participation and use of social media sites would likely aid in understanding the interdependent effects (cf. Hollenbaugh and Ferris, 2014). For example, virtual community motives (e.g., to develop a romantic relationship) might mediate the moderated effect of extraversion and partnership status on social media site membership. It might be the case that the multiplicative effect of extraversion and partnership status on social media membership is explained, in part, by virtual community motives—a measure that directly assesses an approach orientation to online relationships. Such motives represent a more contextualized aspect of what is represented by the interaction of the broad domain of extraversion and the presence/absence of a romantic partner.

Taken together, the results of the present study suggest the utility of personality system frameworks for the study of participation and behaviors in mediated social contexts, especially models that emphasize interdependent relations among traits (e.g., Van Egeren, 2009; DeYoung, 2015). More concretely, the findings suggest the important role of the extraversion domain—the approach drive—in social media site participation and related self-disclosure. Consistent with research showing the importance of greater general activity levels for healthy aging and well-being (e.g., Menec, 2003; Adams et al., 2011; Bogg and Slatcher, 2015), the results show how greater extraversion—as well as emotional stability and openness—might help compensate for aging and health-related declines in the context of social media participation by providing another means of feeling connected and staying engaged, despite age-related role shifts (e.g., retirement, children leaving home) or perceived lower functional status. Far from being solely a function of greater education, income, or access, the results of the present study point to the importance of interrelated dispositional (including neuroticism and openness) and functional and social factors (age, health-related limitations, partner status) in the depiction of the prevalent, but complex, phenomenon of social media participation.

## Ethics statement

The Institutional Review Board of Wayne State University determined that the research reported herein qualified for exemption according to paragraph #2 of the U.S. Department of Health and Human Services Code of Federal Regulations [45 CFR46.101(b)].

## Author contributions

TB designed and commissioned the data collection, conducted the analyses, and wrote the manuscript.

### Conflict of interest statement

The author declares that the research was conducted in the absence of any commercial or financial relationships that could be construed as a potential conflict of interest.
