# Supplementary material for: Social Media Membership, Browsing, and Profile Updating in a Representative U.S. Sample: Independent and Interdependent Effects of Big Five Traits and Aging and Social Factors
Source: Front Psychol. 2017 Jun 30;8:1122. doi: 10.3389/fpsyg.2017.01122 (PMC5492447; doi:10.3389/fpsyg.2017.01122)
Supplement: Supplementary file 1 [file Table1.PDF]

Supplementary Table.

Main and interaction logistic regression effects of extraversion, neuroticism, openness, age, health-related physical limitations, and relationship status on likelihood of social media site membership (N = 992).

	Likelihood of membership (odds ratio)		
	Model 1	Model 2	Model 3
	(Cox-Snell R^2 = .159)	(Cox-Snell R^2 = .163)	(Cox-Snell R^2 = .161)
	OR (95 % CI)	OR (95 % CI)	OR (95 % CI)
Sex	1.98* (1.45, 2.66)	2.04* (1.50, 2.76)	1.96* (1.45, 2.65)
Age	.76* (.64, .90)	.76* (.64, .89)	.76* (.64, .90)
Ethnicity	1.19 (.86, 1.64)	1.12 (.86, 1.64)	1.23 (.89, 1.70)
Education	1.38* (1.16, 1.64)	1.40* (1.17, 1.67)	1.48* (1.24, 1.76)
Household income	.92 (.76, 1.11)	.91 (.76, 1.10)	.89 (.74, 1.08)
Working	1.67* (1.20, 2.31)	1.61* (1.16, 2.23)	1.63* (1.18, 2.25)
Household Internet access	2.80* (1.94, 4.02)	2.79* (1.94, 4.01)	2.85* (1.98, 4.11)
Married/Living with a partner	.93 (.68, 1.28)	.94 (.68, 1.30)	.95 (.68, 1.32)
Health status	.85 (.72, 1.02)	.87 (.73, 1.04)	.84 (.70, 1.00)
Health-related physical limitations	.81† (.67, .99)	.76* (.62, .94)	.82 (.67, 1.00)
Extraversion	1.36* (1.15, 1.61)	1.38* (1.17, 1.64)	1.79* (1.39, 2.31)
Neuroticism	1.22† (1.02, 1.46)	1.20 (1.00, 1.44)	1.25 (.97, 1.62)
Conscientiousness	.89 (.74, 1.06)	.90 (.76, 1.07)	.90 (.76, 1.08)
Agreeableness	1.11 (.93, 1.32)	1.12 (.94, 1.34)	1.08 (.91, 1.29)
Openness	1.08 (.91, 1.28)	1.10 (.93, 1.30)	1.12 (.94, 1.32)

Model 1	OR (95 % CI)	Model 2	OR (95 % CI)	Model 3	OR (95 % CI)
N x O	1.08 (.93, 1.26)	N x E	1.12 (.96, 1.30)	N x E	.94 (.76, 1.17)
N x Age	.86 (.73, 1.01)	N x Limitations	.98 (.77, 1.13)	N x With partner	.94 (.69, 1.29)
O x Age	1.21† (1.03, 1.43)	E x Limitations	.93 (.82, 1.17)	E x With partner	.64* (.46, .88)
N x O x Age	1.09 (.92, 1.29)	N x E x Limitations	.71* (.59, .86)	N x E x With partner	1.26 (.94, 1.68)

Note. *$p < .01$, † $p < .05$. E = Extraversion, N = Neuroticism, O = Openness.
